# Correction: Vassallo et al. Hyaluronic Acid-Based Injective Medical Devices: In Vitro Characterization of Novel Formulations Containing Biofermentative Unsulfated Chondroitin or Extractive Sulfated One with Cyclodextrins. *Pharmaceuticals* 2023, *16*, 1429

**DOI:** 10.3390/ph16121685

**Published:** 2023-12-04

**Authors:** Valentina Vassallo, Celeste Di Meo, Giuseppe Toro, Alberto Alfano, Giovanni Iolascon, Chiara Schiraldi

**Affiliations:** 1 Department of Experimental Medicine, Section of Biotechnology, University of Campania “Luigi Vanvitelli”, 80138 Naples, Italy; valentina.vassallo@unicampania.it (V.V.); celeste.dimeo@unicampania.it (C.D.M.); alberto.alfano@unicampania.it (A.A.); 2 Department of Medical and Surgical Specialties and Dentistry, University of Campania “Luigi Vanvitelli”, 80138 Naples, Italy; giuseppe.toro@unicampania.it (G.T.); giovanni.iolascon@unicampania.it (G.I.)

In the original publication [1], there were mistakes in Figure 1 caption and the location of some software companies. 

## 1. Error in Figure 1 Caption

Chondroitin sulfated should be **chondroitin sulfate**.

The corrected **“Figure 1 caption”** appears below.

Figure 1. Mechanical spectra of the preparations as commercialized. HHA + CS + cd: hyaluronic acid sodium salt 2% (*w*/*v*), marine chondroitin sulfate 2% (*w*/*v*), and cyclodextrins 1% (*w*/*v*); HHA/BC: hyaluronic acid sodium salt 2.4% (*w*/*v*) and unsulfated chondroitin 1.6% (*w*/*v*).

## 2. Errors in Software Company Locations

IBSA Farmaceutici (Lodi, CA, USA) should be **IBSA Farmaceutici, Lodi, Italy;** Kolinpharma (Lodi, CA, USA) (Kolinpharma) should be **Kolinpharma, Milan, Italy;** BD Falcon (BD, Falcon, USA) should be **BD, Falcon, Franklin Lakes, NJ, USA;** Bio-Rad (Beckman, Milano, Italy) should be **Beckman, Milan, Italy;** Company (Bio-Rad, Milan, Laboratories) should be **Bio-Rad Laboratories, Milan, Italy;** Thermo Fisher Scientific (ThermoFisher Scientific, USA) should be **Thermo Fisher Scientific, Waltham, MA, USA.**

The corrected contents appears below.

**Paragraphs 1 and 2 of Section 4.1*. Class III Medical Device Based on HA and CS or BC***:

Sinogel^®^ 3 mL (IBSA Farmaceutici, Lodi, Italy) (here referred to as HHA/BC) was kindly provided by IBSA Farmaceutici Italia.

Dolatrox^®^ (Kolinpharma, Milan, Italy) (here referred to as HHA + CS + cd) was bought in a pharmacy; it is reported to contain hyaluronic acid sodium salt 2% (*w*/*v*), chondroitin sulfate 2% (*w*/*v*), and cyclodextrins 1% (*w*/*v*).

**Paragraphs 1 of Section 4.4*. OA In Vitro Model Setup***:

The next day, the cell suspension was filtered (70 μm, BD Falcon, Franklin Lakes, NJ, USA), centrifuged at 1500 rpm for 7 min (Eppendorf Centrifuge), washed with PBS, and re-centrifuged.

**Paragraphs 1 of Section 4.5*. Cellular Viability Assay***:

The optical densities of the supernatants were measured at 450 nm using a Beckman DU 640 spectrometer (Beckman, Milan, Italy) after specific treatments and incubation with a cell counting solution.

**Paragraphs 1 of Section 4.6*. Gene Expression Analyses via qRT-PCR***:

The variation of gene expression was calculated by normalizing the data about GAGs treated cells with respect to pCTRL, applying the Livak method (2^−ΔΔCt^) [55] and using Bio-Rad iQ™5 Optical System Software, Version 2.1 (Bio-Rad Laboratories, Milan, Italy).

**Paragraphs 1 of Section 4.7. *Protein Analyses via Western Blotting***:

Primary chondrocytes were seeded in a 12-well standard plate (BD Falcon, Franklin Lakes, NJ, USA) (30 × 10^3^ cells/well) and in vitro cultivated in the presence or not (pCTRL) of GAGs-based gels for 7 days at a final concentration in the culture medium of 4 mg/mL.

**Paragraphs 1 of Section 4.8. *Immunofluorescence Analyses***:

To analyze the protein expression of COLII via immunofluorescence staining (IF), primary chondrocytes were seeded in a chamber slide (BD Falcon, Franklin Lakes, NJ, USA) (5 × 10^3^ cells/well) and in vitro cultivated in the presence or not (pCTRL) of GAG-based medical devices (4 mg/mL) for 24 h.

Then, the slices were incubated with a FITC-conjugated goat anti-rabbit secondary antibody (Thermo Fisher Scientific, Waltham, MA, USA) (diluted 1:200) for 1 h at room temperature and covered using the mounting medium with DAPI-aqueous.

**Paragraphs 1 of Section 4.9*. ELISA Assay***:

Each experiment was performed in triplicate, and the cytokine was quantified by a microplate reader (Bio-Rad Laboratories, Milan, Italy).

The authors state that the scientific conclusions are unaffected. This correction was approved by the Academic Editor. The original publication has also been updated.
